# A Pilot of a Simulation-Based Curriculum for Teaching Cardiac Point-of-Care Ultrasound to Internal Medicine Residents

**DOI:** 10.7759/cureus.117735

**Published:** 2026-10-10

**Authors:** Patricia E Simmer, J. Daniel Hess, Susan Coffey-Zern, William Marshall, Richard Baker, Michael T Vest

**Affiliations:** 1 Emergency/Internal Medicine, Christiana Care Health System, Wilmington, USA; 2 Emergency/Internal Medicine, Christiana Care Health System, Newark, USA; 3 Virtual Education and Simulation Training Center, Christiana Care Health System, Newark, USA; 4 Internal Medicine/Critical Care, Sidney Kimmel Medical College, Philadelphia, USA; 5 Internal Medicine/Critical Care Medicine, Christiana Care Health System, Newark, USA

**Keywords:** cardiac pocus, cardiology cardio intervention cardiac imaging gastroenterology internal medicine, internal medicine residency, medical education, pocus (point-of-care ultrasound), simulation training, ultrasound (u/s)

## Abstract

Background: Point-of-care ultrasound (POCUS) as a tool for evaluating patients at bedside is increasingly used in the field of internal medicine. Expectations for internal medicine clinician competency are growing. However, ultrasound is not currently well integrated into graduate medical education for internal medicine residents. Published curricula describe time and resource intensive courses that may be impractical for teaching a broad clinical skill. Further, assessment in prior studies frequently relies on self-reported surveys rather than direct evaluation. We aimed to determine the feasibility of a brief ultrasound curriculum utilizing self-study and simulation-based assessment of image acquisition as well as image interpretation and clinical application, and identify any factors that may affect assessment scores, including clinical exposure.

Methods: In 2024, internal medicine residents (post-graduate-year two and above) were provided with self-study videos on cardiac ultrasound technique and pathology. Residents then participated in a brief hands-on session using live models and high-fidelity ultrasound simulators. They were encouraged to practice during clinical rotations. Participants later returned for testing using a combined high-fidelity ultrasound and patient simulator. Performance was assessed using checklists adapted from published tools. Demographics and prior ultrasound experience were collected by survey.

Results: 10 out of 12 participants completed training, testing, and the survey. Residents performed well in image acquisition, but frequently lost points on image interpretation and clinical management. Residents reporting ≥ 10 clinical echocardiograms performed between testing and training achieved higher mean scores than those with <10 (79.6% vs 57.3%). Prior ultrasound exposure was variable, but generally limited.

Conclusion: The brief curriculum combining self-study with hands-on instruction facilitated internal medicine resident training in bedside cardiac ultrasound with reasonably low use of resources. Brief training may support developing image acquisition skills, but ongoing clinical experience may be important to promote accurate interpretation and application. Self-study resources and simulation have potential to contribute to education and assessment.

## Introduction

Point-of-care ultrasound (POCUS) for bedside patient evaluation is increasingly common in internal medicine clinical practice. Accordingly, graduating internal medicine residents are expected have familiarity with this tool. The American Journal of Medicine has now published the first consensus recommendations for POCUS in internal medicine training [1], formalizing expectations for graduating residents. Although demand for POCUS training in internal medicine residency has been growing for years [2,3], there remains heterogeneity across programs in terms of level of exposure or formal instruction. Growing expectations will push residency programs to incorporate POCUS into their curricula to graduate residents who are competent in this skill.

Published curricula describe time and resource intensive courses, ranging from 30-hour courses to shorter, individual training periods [2,4]. These may be impractical for residency programs to implement. Further, many courses focus only on image acquisition or structure identification, rather than use in clinical decision making and recognizing pathology [4,5]. Internal medicine curricula geared toward competence in clinical applications are sparse in the literature. Those that do focus on clinical competency rely on surveys measuring learners’ comfort with POCUS without objective assessment [5]. Surveys assessing comfort generally correlate poorly with competency [6]. Therefore, there remains a gap in the literature when it comes to efficient curricula that assess clinical competence. Simulation is a useful tool both for training and assessment of competency in graduate medical education [7].

We aimed to determine the feasibility of a brief cardiac POCUS curriculum utilizing self-study and simulation-based assessment of image acquisition as well as image interpretation and application in three clinical scenarios: cardiac tamponade, distributive shock with hyperdynamic ejection fraction (EF), and cardiogenic shock. We also collected survey information to identify factors that may affect assessment scores, including clinical exposure.

## Materials and methods

Curriculum and evaluation design

For this pilot we focused on cardiac POCUS, because it is validated for evaluating patients with a variety of presentations (shortness of breath, shock, etc.) [8-10]. Further, we predicted that opportunity for clinical exposure to cardiac POCUS would be high compared to other exams, allowing residents to gain repeated clinical exposure between curriculum didactics and skills assessment. A primary aim was practicality, so self-study resources were incorporated, followed by hands-on instruction sessions. For evaluation, we created high-yield simulated scenarios based on common clinical situations where cardiac POCUS would be an appropriate tool to use within the scope of an internal medicine clinician. We designed an evaluation tool based on examples in the literature [11-15]. Authors refined the tool to make a second iteration which decreased the number of points allotted to specific views and clinical actions, and increased points allotted to overall success in obtaining adequate images to make the appropriate diagnosis, and interpretation of clinically relevant findings. This change was made in the attempt to distinguish those residents competent in POCUS from those still developing the skill. Final scoring checklists in Tables 1, 2, 3.

**Table 1:** Scoring checklist for tamponade scenario Total number of possible points: 100; Passing score: 70 IVC: Inferior vena cava; EF: Ejection fraction; RV: Right ventricle; LV: Left ventricle

Tamponade Scenario: Obtains following images and correctly identifies anatomy	Yes	No	Comment
Subxiphoid image with appropriate depth to visualize entire pericardium	2.5	0	
IVC view	2.5	0	
Parasternal long axis view	2.5	0	
Parasternal short axis view	2.5	0	
Apical 4-chamber view with adequate visualization of all 4 chambers	2.5	0	
Views of adequate quality for diagnosis	25	0	
Correctly Assesses	Yes	No	Comment
Presence or absence of reduced EF	10	0	
Comparison of size of RV and LV	2.5	0	
Presence or absence of pericardial effusion	25	0	
Diagnosis and Treatment	Yes	No	Comment
Verbalizes concern for tamponade aloud - 10 points; Verbalize concern for shock with pericardial effusion - 5 points	10	0	
Appropriately manages shock with pressors and fluids	10	0	
Stat call to interventional cardiology about concern for tamponade	5	0	

**Table 2:** Scoring checklist for sepsis/distributive shock with hyperdynamic EF scenario Total number of possible points: 100; Passing score: 70 IVC: Inferior vena cava; EF: Ejection fraction; RV: Right ventricle; LV: Left ventricle; CHF: Congestive heart failure

Sepsis/Distributive Shock Scenario: Obtains following images and correctly identifies anatomy	Yes	No	Comment
Subxiphoid image with appropriate depth to visualize entire pericardium	2.5	0	
IVC view	2.5	0	
Parasternal long axis view	2.5	0	
Parasternal short axis view	2.5	0	
Apical 4-chamber view with adequate visualization of all 4 chambers	2.5	0	
Views of adequate quality for diagnosis	25	0	
Correctly Assesses (must verbalize assessment)	Yes	No	Comment
Recognizes hyperdynamic heart with absence of severely reduced EF: hyperdynamic - 30 points; normal/not reduced - 20 points; reduced EF - 0 points	30	0	
Comparison of size of RV and LV	2.5	0	
Presence or absence of pericardial effusion	5	0	
Diagnosis and Treatment	Yes	No	Comment
Articulates diagnosis of septic shock or distributive shock: septic shock - 10 points; undifferentiated shock - 5 points; cardiogenic shock/CHF - 0 points	10	0	
Appropriate fluids and vasopressors to manage shock	10	0	
Call to medical ICU about concern for septic shock	5	0	

**Table 3:** Scoring checklist for cardiogenic shock scenario Total number of possible points: 100; Passing score: 70 IVC: Inferior vena cava; EF: Ejection fraction; RV: Right ventricle; LV: Left ventricle; CHF: Congestive heart failure

Cardiogenic Shock Scenario: Obtains following images and correctly identifies anatomy	Yes	No	Comment
Subxiphoid image with appropriate depth to visualize entire pericardium	2.5	0	
IVC view	2.5	0	
Parasternal long axis view	2.5	0	
Parasternal short axis view	2.5	0	
Apical 4-chamber view with adequate visualization of all 4 chambers	2.5	0	
Views of adequate quality for diagnosis	25	0	
Correctly Assesses	Yes	No	Comment
Recognizes severely reduced EF	25	0	
Comparison of size of RV and LV	2.5	0	
Presence or absence of pericardial effusion	5	0	
Diagnosis and Treatment	Yes	No	Comment
Articulates diagnosis: cardiogenic shock - 15 points; CHF - 5 points; other diagnosis - 0 points	15	0	
Gives pure alpha agonist or large fluid bolus >1 liter - 0 points; Gives appropriate pressors for cardiogenic shock - 10 points	10	0	
Call to cardiology and/or ICU about concern for cardiogenic shock - 5 points	5	0	

Setting and participants

Residents who had completed post-graduate-year one and were in either categorical internal medicine or combined internal medicine/pediatrics programs were eligible for participation. All eligible residents in the cohort (n=36) were invited to participate on a voluntary basis. 10 residents ultimately participated and completed the entire curriculum. As this was a feasibility study, sample size was determined by the number of eligible residents available during the study period, and no a priori power calculation was performed. All residents had prior exposure to POCUS for procedural guidance when receiving formal training for central line and arterial line placement in the simulation center. Residents had opportunity to use POCUS clinically during inpatient rotations and emergency medicine rotations.

Implementation of curriculum

Residents were provided self-study videos describing cardiac POCUS technique and identifiable pathology. After reviewing those resources [16,17], they participated in a two-hour, hands-on instruction session led by one or two attending physicians credentialed in POCUS (one internal medicine/emergency medicine and one pulmonary/critical care). Each session was limited to four learners maximum. During the session, instructors went through all four views of the cardiac POCUS exam, as well as the inferior vena cava (IVC) view. They instructed residents on how to troubleshoot with the ultrasound machine to optimize images, take measurements, and save clips or still images. Residents practiced on both a high-fidelity ultrasound simulator (CAE Vimedix Ultrasound Simulator) and live models until they demonstrated ability to obtain adequate views. Images from the session were saved on a commercial ultrasound image archiving and review platform (QpathE) to reinforce the process and importance of saving images. After this session, residents were encouraged to practice and seek attending feedback while on clinical rotations.

Assessment and evaluation

After at least one month, residents returned for follow-up evaluation where a combined high-fidelity ultrasound simulator (CAE Vimedix Ultrasound Simulator) and patient simulator (CAE Metiman) challenged them with three simulated scenarios over the course of one hour: one case of cardiac tamponade with a large pericardial effusion; one of septic shock with hyperdynamic EF; and one of cardiogenic shock with severely reduced EF. Details on simulation scenarios and setup are included in Appendix 1, 2, and 3. The sessions were proctored by at least one simulation staff member and one faculty instructor. Sessions were recorded, allowing for proctors to review and complete checklist assessments after the fact, even if they weren’t originally present for assessment. After assessment, residents completed a survey of demographic information and POCUS experience both prior to training and in the interim between curriculum and evaluation. The survey instrument is shown in Figure 1. After iteration of the scoring checklists, a single team member reviewed simulation footage and scored each with the updated tool. These results were used in the final analysis.

**Figure 1:**
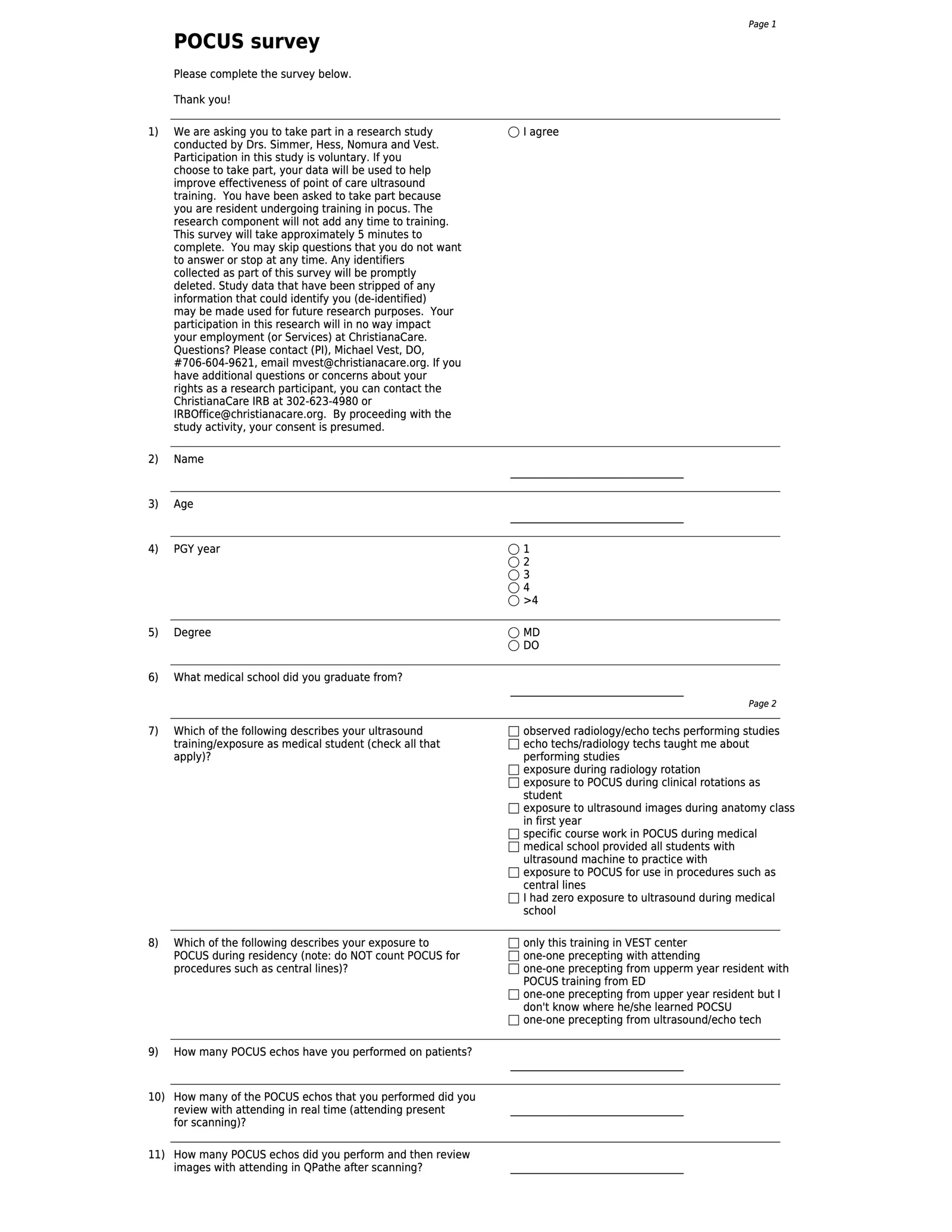
Survey instrument

Data analysis

Data were summarized using descriptive statistics. Assessment scores for image acquisition versus interpretation were compared using a paired two-tailed t-test. Because of the small sample size, a Wilcoxon signed-rank test was also performed. Statistical significance was defined as a two-sided p value <0.05. Residents were stratified according to their self-reported clinical POCUS experience between the time of training and assessment. Comparisons between these groups were performed using Welch's two-sample t-test due to unequal sample sizes and variance between groups.

This study was reviewed by our institutional review board (IRB00000480; FWA00006557) and deemed exempt on 11/15/2023 (DDD# 605464).

## Results

Between February and September 2024, a total of 12 residents participated in at least one aspect of the study. 10 residents completed training, simulated testing scenarios, and post-training survey: 83.3% of original learners, suggesting good learner engagement. Data from these 10 residents were used in final analysis. Performance was variable. Average scores across all scenarios ranged from 26.7% to 86.7% (mean, 64.0%; median, 69.2%; SD, 19.6). Residents performed best in the tamponade scenario (mean, 68.4%; median, 71.3%; SD, 21.3).

There was a non-statistically-significant trend toward higher scores on image acquisition compared to interpretation and clinical application. Mean score for image acquisition was 71.8% (SD, 26.8), and 59.4% (SD, 18.2) for interpretation and application overall (paired t-test, t(9) = -2.01, p=0.0756). A Wilcoxon signed-rank test yielded similar results (W = 10, p = 0.07).

Prior exposure to diagnostic (rather than procedural) POCUS was variable but generally limited. Survey results regarding prior POCUS exposure is summarized in Figure 2. In the time between training and evaluation, residents reported performing between zero and 15 clinical POCUS transthoracic echocardiograms (pTTE). Residents who had performed ≥10 pTTEs in that time had higher overall scores (mean 79.6%; SD 10.5 vs. mean 57.3%; SD 19.1; Welch’s t-test, t(6.99)=2.37, p=0.0499). The statistical difference in scores persisted when examining interpretation and clinical application in the group who performed ≥10 pTTEs (74.4%; SD 6.0%) vs those who performed fewer than 10 (53.0%; SD 17.9%; Welch’s t-test, t(7.93)=2.82, p=0.0226). However, there was no statistical difference between groups in scores for image acquisition alone (≥10 pTTEs mean 88.1%; SD 18.6 vs. <10 pTTEs mean 64.8%; SD 27.8; Welch’s t-test, t(5.85)=1.56, p=0.172). See Figure 3. Table summary of these data is also available in Appendix 4.

**Figure 2:**
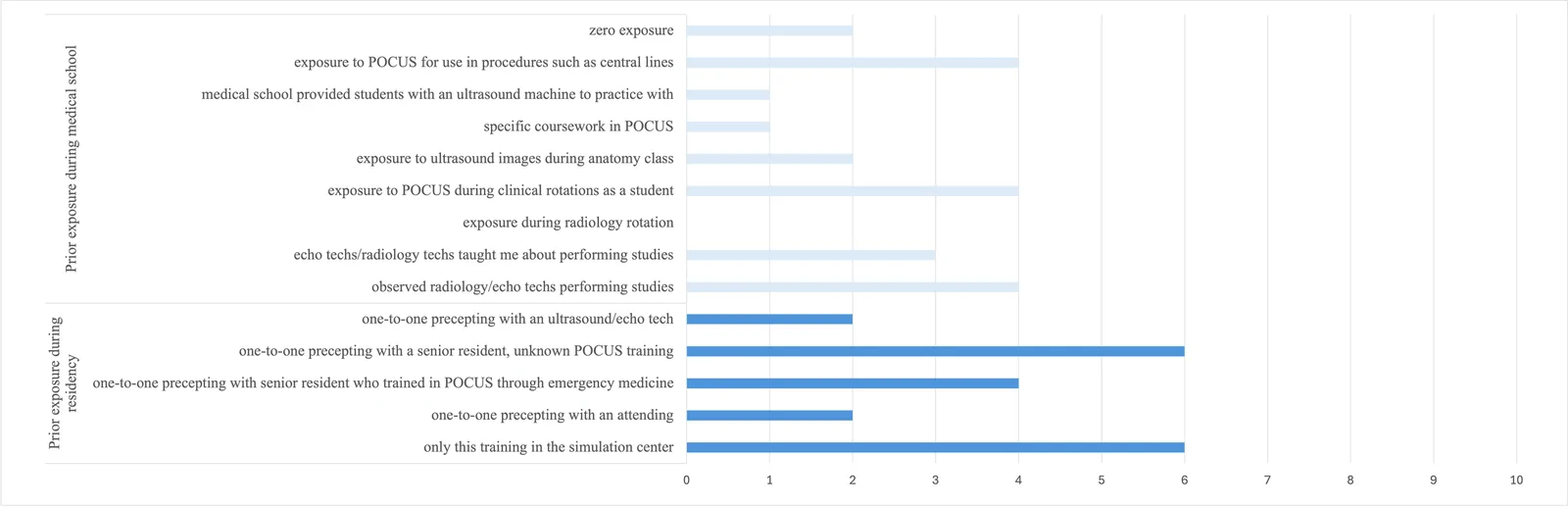
Survey data - prior exposure to ultrasound training in medical school and residency

**Figure 3:**
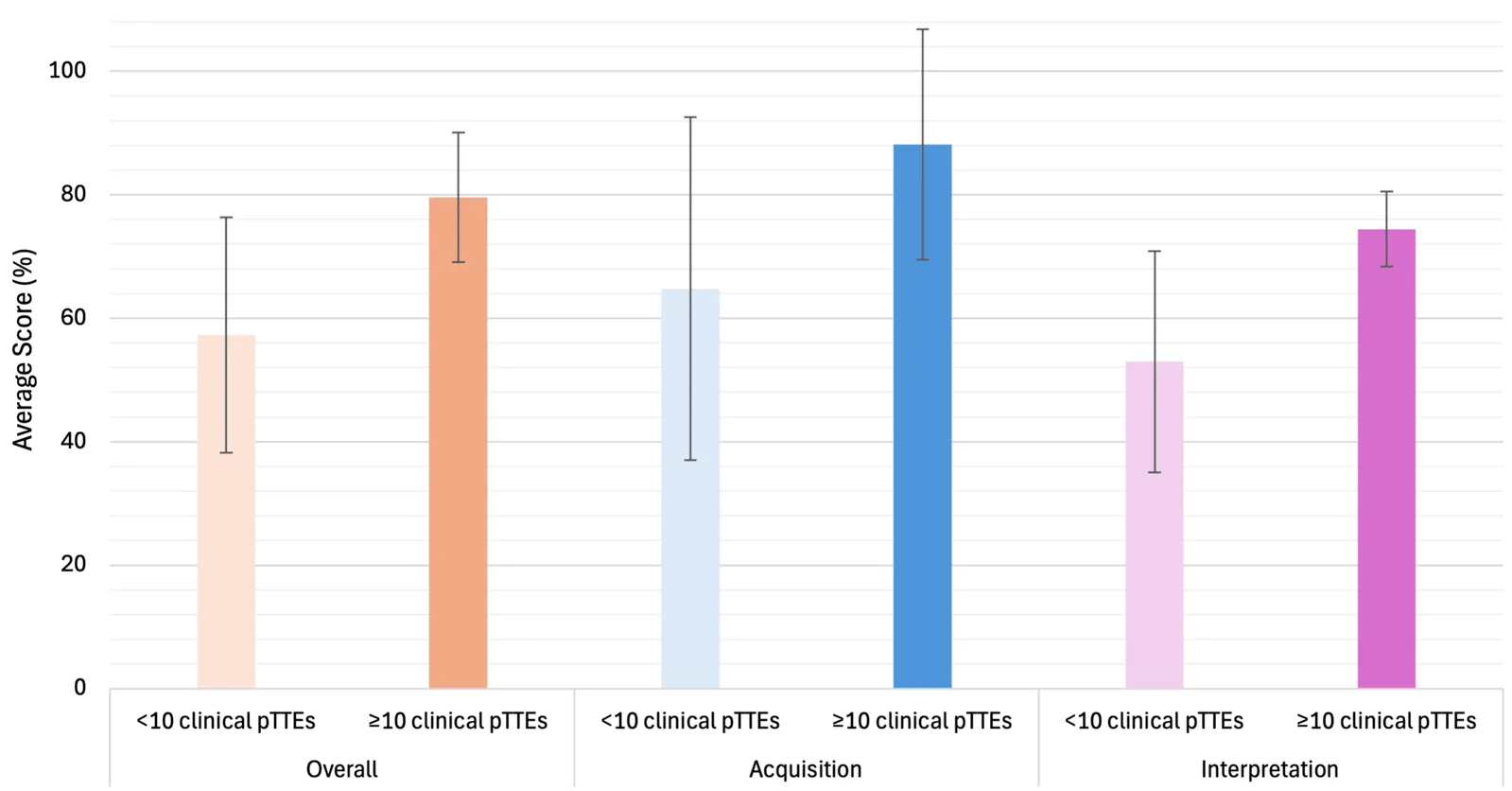
Average scores (%) overall, for image acquisition only, and for interpretation and clinical application only; stratified by clinical pTTEs performed between training and testing Mean scores on simulated clinical scenario testing, shown as overall performance and performance on the acquisition portion versus the interpretation/clinical application portion. Compared using a paired two-tailed t-test and a Wilcoxon signed-rank test. Data are stratified by number of clinical bedside pTTEs performed between the instruction session and testing session (<10 or ≥10). Comparisons of these groups were performed using Welch's two-sample t-test. pTTE: Point-of-care transthoracic echocardiogram

There were between four and five person-hours required to prepare and test-run scenarios. Training and assessment of four residents took approximately six person-hours of faculty time, and four person-hours of simulation center/instructor time.

## Discussion

A short curriculum for cardiac POCUS incorporating self-study and hands-on instruction, followed by evaluation using simulation showed feasibility as estimated by high percentage of participants who completed the entire course, and reasonably low use of resources. Overall, scores on image acquisition were high. Interpretation and clinical application scores were lower, but seemed to improve with clinical practice.

A 2025 consensus statement summarizes expert and evidence-based recommendations for internal medicine core competencies in POCUS, identifying 52 specific skills [1]. Cardiac POCUS recommendations specify the four views: parasternal long axis, parasternal short axis, apical four chamber, and subcostal four chamber, which were all included in our curriculum (we also included IVC evaluation). The consensus statement also recommends trainees be able to evaluate for qualitative left ventricular function (assess normal, hyperdynamic, and severely reduced left ventricular systolic function), qualitatively assess the IVC to help inform volume status exams, and evaluate for presence of a pericardial effusion. These align well with previously published guidelines [8], and were all targeted in our curriculum and addressed in evaluations.

After intervention, residents demonstrated ability to obtain appropriate views for pTTE. However, due to lack of a control group or pre-intervention assessment, formal evaluation of the effectiveness of the curriculum could not be performed. Further, we found clinical experience was associated with improved scores overall. However, this was a result observed among multiple comparisons, and not tied to an original aim or hypothesis of the study. No adjustment for multiple comparisons was performed. It is accepted that metrics beyond simple number of studies performed are needed to assess learner competence [18]. Simulation has the potential to play an important role in both training and assessment of POCUS skills.

Our study is limited in that it is a single center study with few participants. Although we referenced validated tools when creating our assessments, our iterations were not validated. The number of learners in each training session was variable, a factor that could have influenced their learning/performance. There was some risk for bias in our study design. Although survey results weren’t analyzed until after all assessments and scores were completed, there was no official blinding protocol, and the scorer may have been biased if they had information regarding a resident’s prior ultrasound exposure, or clinical experience in the interim between training and testing. Our results were also subject to confounders. For example, those who performed more clinical exams between curriculum and testing who scored higher on interpretation and clinical application may have had higher intrinsic motivation, or even had more prior exposure, increasing their comfort to perform more bedside exams on clinical rotations. We also relied on self-reporting for number of clinical exams performed. In the future, review of saved clinical images, confirmation of completion, and assessment of quality and interpretation will improve the curriculum.

Given the growing availability of ultrasound as a tool for internal medicine clinicians, and expectation that graduating residents will have the skills to use it clinically, it will be important to find efficient and feasible means to ensure trainees are prepared to integrate POCUS into patient care and medical decision making. Validation of evaluation tools, as well as investigation comparing resident performance both before and after participation in curriculum should be pursued.

## Conclusions

A brief curriculum combining self-study with hands-on instruction facilitated internal medicine resident training in pTTE with high level of participant completion. Training and assessment using high-fidelity simulation utilized an acceptable amount of resources to implement. Results of resident evaluation suggested that all learners were able to score well on image acquisition after participation. Although, without a control group or pre-intervention evaluation, we can’t draw conclusions on whether this was a result of the curriculum itself. There was a signal that increased clinical experience was associated with higher scores in image interpretation and clinical application scores, although confounders and bias could have influenced this result. The study was limited by sample size, and lack of assessment tool validation. Future investigation validating assessment tools, and incorporating a control group or pre-intervention assessment, and with larger numbers of participants should be pursued.

## Appendices

Appendix 1

**Table 4:** POCUS testing tamponade scenario - simulation setup checklist POCUS: Point-of-care ultrasound; BP: Blood pressure; SBAR: Situation, Background, Assessment, and Recommendation; WBC: White blood cell count (cells/microL); Hgb: Hemoglobin (g/dL); PLT: Platelet count (/microL); K: Potassium (meq/L); CO_2_: Bicarbonate level (meq/L); BUN: Blood urea nitrogen (mg/dL)

Tamponade Scenario			
Clinical/Medical Learning Objective(s): Use POCUS to identify pericardial effusion and manage patient with obstructive shock			
Simulation Team/Facilitator(s)/Standardized Patients(s): Manikin, ultrasound simulator Simulation Center Staff to Run Simulator: Vest or Hess or Simmer			Learner(s): Medicine Residents
Brief Session Description: 55-year-old woman with history of breast cancer admitted to the oncology ward. Rapid response team called for shortness of breath. Initial vitals: Heart rate 120, BP 80/60, oxygen saturation 87% on room air.			
Scenario Room and Equipment Setup: Manikin CAE Vimedix Ultrasound simulator set to Tamponade; Debrief Room and equipment setup: (2 Rooms)			
Patient Background			
Patient name: Lola Age: 55			
SBAR, Hand Off or Door Sign: S: 55-year-old woman admitted for work-up of breast cancer. She is scheduled for staging CT scans, which have not yet been done. She was a little short of breath at beginning of shift, but during report to oncoming shift, it was noted that she was much worse, sitting in tripod position with increased work of breathing. Rapid response team called. B: New diagnosis of breast cancer. Hasn’t received any treatment yet. A: Severe dyspnea, R: Bedside nurse reports patient probably needs to be transferred to ICU.			
Interview of Patient: (This information is what the simulation center staff operating the manikin should know and say if asked about their present illness and medical history. Areas in quotes should be stated verbatim.) Patient feels very short of breath and lightheaded. She does not feel palpitations. She has had constant chest pain for a long time and is used to it. She noted hard lump on right breast couple of years ago. However, she doesn’t like doctors and didn’t seek care. Daughter noticed it couple of weeks ago and made her go to doctor. Biopsy showed adenocarcinoma. Oncologist admitted her to facilitate staging work-up and maybe start chemotherapy.			
Simulation Pathway			
	Learner Actions	Response to Learner Actions	
Learners Enter Room	Brief history above, physical unremarkable	No change	
Assessment	Recognizes shock - initiates some therapy	If orders IV fluids, BP improves. If orders vasopressors, BP improves but tachycardia worsens. If does not order either fluids or vasopressors, then BP decreases and heart rate increases.	
	POCUS - tamponade setting on CAE Vimedex -> Recognizes pericardial effusion and calls for stat cardiology consult	Note: Not expected to diagnose tamponade, but clinically suspect. Must call cardiologist directly to discuss emergency – must articulate suspicion for tamponade – if does this and fluid not already given, cardiologist recommends IV fluid to temporize while coming to help -> scenario ends	
	POCUS - tamponade setting on CAE Vimedex -> Recognizes pericardial effusion and resident plans pericardiocentesis themselves	Scenario ends -> no recommended action outside of code	
	POCUS - tamponade setting on CAE Vimedex -> resident fails to recognize effusion OR recognizes effusion but fails to call for emergent help (e.g. plan is something vague like transfer to ICU)	BP and heart rate worsen -> if calls cardiology emergently then see above; if fails to call for emergent help -> scenario ends	
Lab Results and Other Studies:			
PowerPoint displayed on screen. If technical errors arise, findings can be read overhead, or printouts of slides can be provided to the learners. Morning labs: WBC 6, Hgb 14, PLT 300, Creatinine 0.7, K 4.0, CO_2_ 28, BUN 18			
Debriefing			

Appendix 2 

**Table 5:** POCUS testing septic shock with findings of hyperdynamic EF scenario - simulation setup checklist EF: Ejection fraction; POCUS: Point-of-care ultrasound; BP: Blood pressure; SBAR: Situation, Background, Assessment, and Recommendation; HFrEF: Heart failure with reduced ejection fraction; MAP: Mean arterial pressure; WBC: White blood cell count (cells/microL); Hgb: Hemoglobin (g/dL); PLT: Platelet count (/microL); K: Potassium (meq/L); CO_2_: Bicarbonate level (meq/L); BUN: Blood urea nitrogen (mg/dL)

Shock with Hyperdynamic EF Scenario			
Clinical/Medical Learning Objective(s): Use POCUS to identify fluid responsiveness and manage patient with distributive shock (sepsis scenario)			
Simulation Team/Facilitator(s)/Standardized Patients(s): Manikin, ultrasound simulator Simulation Center Staff to Run Simulator: Vest or Hess or Simmer			Learner(s): Medicine Residents
Brief Session Description: 75-year-old man with history of prior stroke with residual dysphagia, admitted to the hospital for suspected aspiration pneumonia. Rapid response team called for hypotension. Initial vitals: Heart rate 120, BP 80/60, oxygen saturation 91% on 2L nasal cannula.			
Scenario Room and Equipment Setup: Manikin CAE Vimedix Ultrasound simulator set to Hyperdynamic Left Ventricular Systolic Function; Debrief Room and Equipment Setup: (2 Rooms)			
Patient Background			
Patient name: Lloyd Age: 75			
SBAR, Hand Off or Door Sign: S: 75-year-old man with history of HFrEF with EF 40-45% on last echocardiogram 6 months ago, prior stroke with residual dysphagia, admitted to the hospital for treatment of suspected aspiration pneumonia. He has been requiring minimal oxygen by nasal cannula, and has received two days of IV antibiotics. His BPs have been low with systolic BP consistently >100 and MAP >70. In the afternoon, the patient was noted to be drowsy and manual BP was low. Rapid response team called for hypotension. Initial vitals: Heart rate 120, BP 80/55, oxygen saturation 91% on 2L by nasal cannula. Extremities are warm and you note bilateral lower extremity edema. B: Suspected aspiration pneumonia. A: Hypotension. R: Bedside nurse reports patient probably needs to be transferred to ICU.			
Interview of Patient: (This information is what the simulation center staff operating the manikin should know and say if asked about their present illness and medical history. Areas in quotes should be stated verbatim.) Patient is drowsy, but arouses to voice. Speaks in 1-2 word sentences. He denies pain or worsening shortness of breath. Patient is diaphoretic. Temperature (if checked) is 35 degrees Celsius.			
Simulation Pathway			
	Learner Actions	Response to Learner Actions	
Learners Enter Room	Brief history above, physical unremarkable	No change	
Assessment	Recognizes shock - initiates some therapy	If orders IV fluids, BP improves. If orders vasopressors, BP improves but tachycardia worsens. If does not order either fluids or vasopressors, both BP decrease and heart rate increases.	
	POCUS - distributive shock setting on CAE Vimedex -> Recognizes hyperdynamic LV	Note: Not expected to measure LVEF, but expected to recognize patient is likely not in cardiogenic shock and will be responsive to fluids. If no fluids given, patient mental status will continue to decline, vasopressor requirements will continue to climb (if didn’t start vasopressors then BP will continue to decrease).	
	POCUS - distributive shock setting on CAE Vimedex -> Does not recognize signs of fluid responsiveness. Suspicion for other types of shock. Initiates inappropriate therapy (such as inotrope)	BP and heart rate worsen -> If calls cardiology emergently then see below; if calls medical ICU then ICU suggests fluids and accepts patient -> scenario ends	
	POCUS - distributive shock setting on CAE Vimedex -> resident fails to recognize fluid responsiveness. Suspicion for other types of shock. Reaches out to inappropriate service (e.g., starting inotrope or calling cardiology).	Cardiology says “it’s not the heart”, will ask about IVC if resident has not yet obtained that view, will suggest consulting medical ICU -> see above	
Lab Results and Other Studies			
PowerPoint displayed on screen. If technical errors arise, findings can be read overhead, or printouts of slides can be provided to the learners. Morning labs: WBC 15, Hb 13, PLT 250, Creatinine 1.5, K 4.0, CO_2_ 32, BUN 24			
Debriefing			

Appendix 3 

**Table 6:** POCUS Cardiogenic Shock Testing Scenario - Simulation Setup Checklist POCUS: Point-of-care ultrasound; EF: Ejection fraction; HFrEF: Heart failure with reduced ejection fraction; SBAR: Situation, Background, Assessment, and Recommendation; BP: Blood pressure; MAP: Mean arterial pressure; IVC: Inferior vena cava; WBC: White blood cell count (cells/microL); Hgb: Hemoglobin (g/dL); PLT: Platelet count (/microL); K: Potassium (meq/L); CO_2_: Bicarbonate level (meq/L); BUN: Blood urea nitrogen (mg/dL)

Cardiogenic Shock Scenario			
Clinical/Medical Learning Objective(s): Use POCUS to identify severely reduced EF and manage patient with cardiogenic shock			
Simulation Team/Facilitator(s)/Standardized Patients(s): Manikin, ultrasound simulator Simulation Center Staff to Run Simulator: Vest or Hess or Simmer			Learner(s): Medicine Residents
Brief Session Description: 75-year-old man with history of HFrEF with EF 40-45% on last echocardiogram 6 months ago, prior stroke with residual dysphagia, admitted to the hospital for suspected aspiration pneumonia. Rapid response team call initiated for hypotension. Initial vitals: Heart rate 120, BP 70/60, oxygen saturation 91% on 2L by nasal cannula.			
Scenario Room and Equipment Setup: Manikin CAE Vimedix Ultrasound simulator set to: Dilated Cardiomyopathy - Very Severe Left; Debrief Room and Equipment Setup: (2 Rooms)			
Patient Background			
Patient name: Lyndon Age: 75			
SBAR, Hand Off or Door Sign: S: 75-year-old man with history of HFrEF with EF 40-45% on last echo 6 months ago, prior stroke with residual dysphagia, admitted to the hospital for treatment of suspected aspiration pneumonia. He has been requiring minimal oxygen by nasal cannula, and has received two days of IV antibiotics. His BPs have been soft with systolic BP consistently >100 and MAP >70. In the afternoon the patient was noted to be drowsy and manual BP was low. Rapid response team called for hypotension. Initial vitals: Heart rate 120, BP 70/60, oxygen saturation 91% on 2L by nasal cannula. Extremities are cool and you note bilateral lower extremity edema. B: Suspected aspiration pneumonia. A: Hypotension. R: Bedside nurse reports patient probably needs to be transferred to ICU.			
Interview of Patient: (This information is what the simulation center staff operating the manikin should know and say if asked about their present illness and medical history.) Patient is drowsy, but arouses to voice. Speaks in 1-2 word sentences. He denies chest pain. If asked if his chest feels “heavy” or “tight” specifically, he’ll say “yes.” Reports slight worsening of shortness of breath. Patient is diaphoretic. Temperature (if checked) is 36.8 degrees Celsius.			
Simulation Pathway			
	Learner Actions	Response to Learner Actions	
Learners Enter Room	Brief history above, physical unremarkable	No change	
Assessment	Recognizes shock and initiates some therapy	If orders 500cc IV fluids, BP improves marginally. If given > 500cc, BP improves marginally, but shortness of breath and oxygen requirement worsens. If orders vasopressors, BP improves. If orders pure alpha agonist, BP improves but shortness of breath and oxygen requirement worsen. If does not order either fluids or vasopressors, BP decreases and heart rate increases.	
	POCUS - cardiogenic shock setting on CAE Vimedex -> Recognizes plump IVC without respiratory variation, poor left ventricular squeeze	Note: Not expected to measure left ventricular EF, but expected to recognize patient has worsened left ventricular EF with poor squeeze. Must call cardiologist directly to discuss emergency, must articulate suspicion for acute cardiogenic shock. If this is done and vasopressors not already started, cardiologist recommends vasopressors to temporize while coming to help. -> Scenario ends	
	POCUS - cardiogenic shock setting on CAE Vimedex -> Does not recognize signs of cardiogenic shock, initiates inappropriate treatment	If giving fluids then work of breathing worsens. If calls medical ICU emergently then see below. If calls cardiology, they suggest stopping fluids and starting vasopressors (if applicable) and accepts patient. -> Scenario ends.	
	POCUS - cardiogenic shock setting on CAE Vimedex -> Resident fails to recognize worsening respiratory status with fluids. Suspicion for other types of shock. Reaches out to inappropriate service (such as calling medical ICU).	Medical ICU will ask about IVC if resident has not yet obtained that view - will suggest calling cardiology (see above). -> Scenario ends	
Lab Results and other Studies			
PowerPoint displayed on screen. If technical errors arise, findings can be read overhead, or printouts of slides can be provided to the learners. Morning Labs: WBC 15, Hgb 13, PLT 250, Creatinine 1.5, K 4.0, CO_2_ 28, BUN 24			
Debriefing			
Debriefing modality, considerations, etc.			

Appendix 4 

**Table 7:** Summary of survey and evaluation data Data for evaluation are given in percentages of possible points earned. Each scenario had 37.5 possible points for image acquisition and 62.5 possible points for image interpretation and clinical application. Highlighted: Residents who performed ≥10 clinical bedside pTTEs between the instruction session and testing session. pTTE: Point-of-care transthoracic echocardiogram

			Simulated Scenario											
Record	Post-graduate Year	# Clinical pTTEs	Cardiogenic Shock			Sepsis			Tamponade			Average Scores		
			Acquisition	Interpretation	Overall	Acquisition	Interpretation	Overall	Acquisition	Interpretation	Overall	Acquisition	Interpretation	Overall
1	3	2	13.3	24.0	20.0	13.3	64.0	45.0	13.3	16.0	15.0	13.3	34.7	26.7
2	4	3	93.3	72.0	80.0	93.3	40.0	60.0	86.7	64.0	72.5	91.1	58.7	70.8
3	3	15	93.3	92.0	92.5	20.0	48.0	37.5	86.7	64.0	72.5	66.7	68.0	67.5
4	2	10	100.0	80.0	87.5	100.0	64.0	77.5	93.3	96.0	95.0	97.8	80.0	86.7
5	2	0	100.0	56.0	72.5	100.0	76.0	85.0	86.7	64.0	72.5	95.6	65.3	76.7
6	2	3	20.0	0.0	7.5	93.3	16.0	45.0	80.0	64.0	70.0	64.4	26.7	40.8
7	2	1	6.7	0.0	2.5	86.7	80.0	82.5	73.3	56.0	62.5	55.6	45.3	49.2
8	2	10	100.0	96.0	97.5	100.0	48.0	67.5	100.0	82.0	88.8	100.0	75.3	84.6
9	2	2	80.0	56.0	65.0	80.0	100.0	92.5	73.3	72.0	67.5	77.8	76.0	75.8
10	2	0	46.7	56.0	52.5	46.7	80.0	67.5	73.3	56.0	62.5	55.6	64.0	60.8
Average		4.6	65.3	53.2	57.8	73.3	61.6	66.0	76.7	63.4	68.4	71.8	59.4	64.0
Median		2.5	86.7	56.0	68.8	90.0	64.0	67.5	83.3	64.0	71.3	72.2	64.7	69.2
StDev		5.2	39.4	34.9	35.7	33.8	24.2	18.9	24.0	20.7	21.3	26.8	18.2	19.6
